# Supplementary figures and images for: Counting cytoplasmic incompatibility factor mRNA using digital droplet PCR
Source: bioRxiv. 2025 Jul 30:2025.07.30.667682. Preprint. [Version 1] doi: 10.1101/2025.07.30.667682 (PMC12324539; doi:10.1101/2025.07.30.667682)

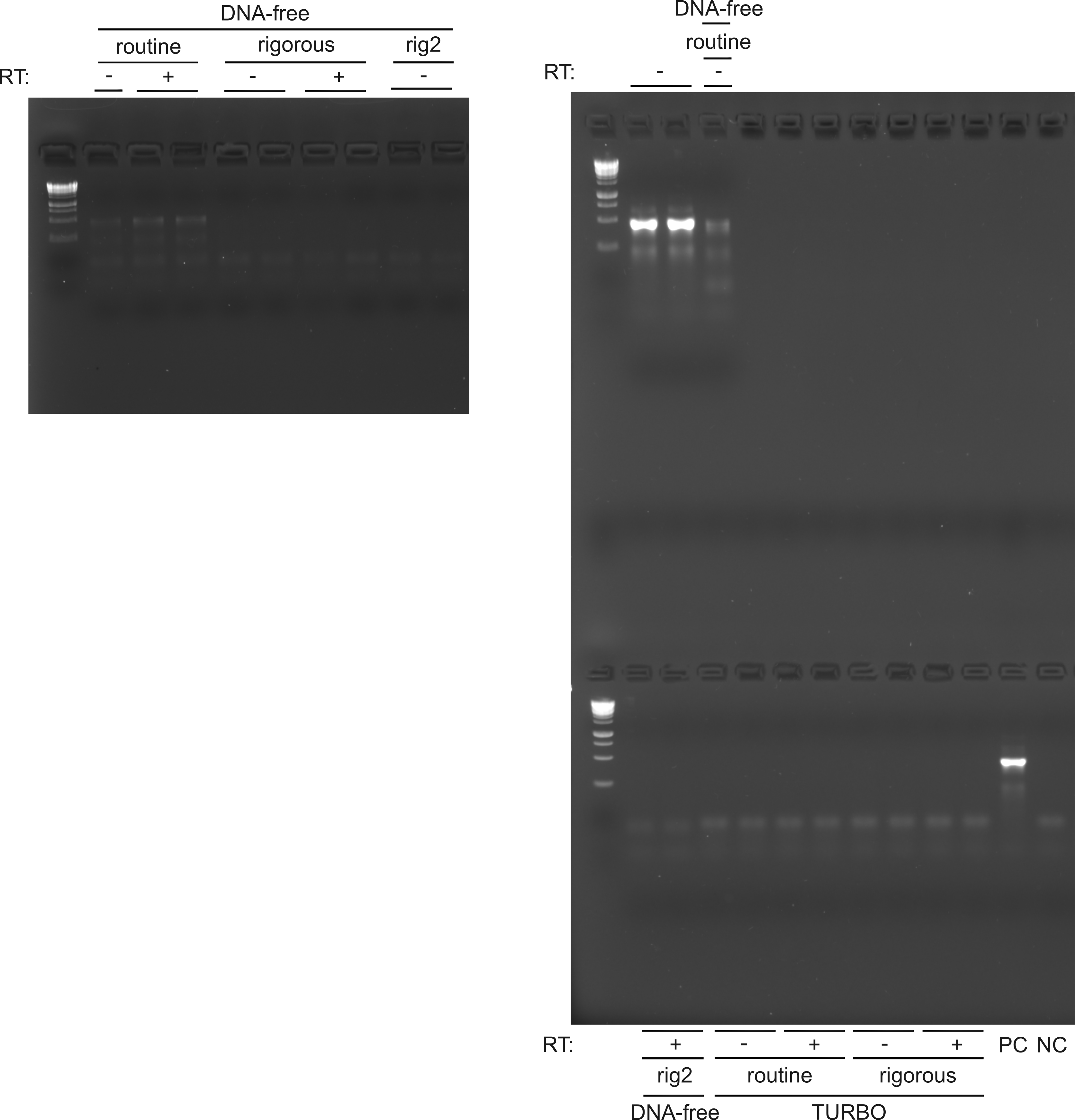

Supplement: Supplement 1 — Data S1. RNA extraction and purification measurements. Data S2. FASTA file containing cifA sequences homologous to the cifA RT-ddPCR oligos. Data S3. FASTA file containing cifB sequences homologous to the cifB RT-ddPCR oligos. Data S4. cifA/spike RT-ddPCR annealing temperature measurements. Data S5. cifB/spike RT-ddPCR annealing temperature measurements. Data S6. cifA/spike RT-ddPCR dilution series measurements. Data S7. cifB/spike RT-ddPCR dilution series measurements. Data S8. cifA/β-Spec RT-ddPCR dilution series measurements. Data S9. cifB/β-Spec RT-ddPCR dilution series measurements. Data S10. DNase treatment raw gel images. Data S11. DNase treatment RT-ddPCR results. [file media-1.zip › Data-S10.png]
